# Supplementary material for: Maternal Vitamin D Levels during Pregnancy and Offspring Psychiatric Outcomes: A Systematic Review
Source: Int J Mol Sci. 2022 Dec 21;24(1):63. doi: 10.3390/ijms24010063 (PMC9820292; doi:10.3390/ijms24010063)

Supplementary Material 1: Search strategy

PsychINFO (via EBSCOhost)

(“Vitamin D*” OR “Vit D*” OR “25-hydroxyvitamin D*” OR “25(OH)D*” OR “25-OH-D*” OR “25 OH D*” OR “1,25-dihydroxyvitamin D*”) AND (pregnancy OR antenatal OR “ante-natal*” OR “ante natal*” OR antepartum OR “ante-partum*” OR “ante partum*” OR pregnant OR prenatal OR “pre-natal*” OR “pre natal*” OR maternal OR mother* OR gestation* OR utero OR delivery OR perinatal OR “peri natal*” OR “peri-natal*” OR offspring* OR child* OR infan* OR newborn* OR baby OR babies OR adolesc* OR toddler* OR preschooler* OR “pre-schooler*” OR “pre schooler*” OR “adult*” OR “early life*”) AND (sera OR biomarker* OR serum OR plasma OR blood OR circulating OR cord) AND (“psychiatric illness*” OR “psychiatric diagnos*” OR “psychiatric disorder*”OR “mental disorder*” OR “mental health*” OR “mental illness*” OR schizo* OR psychos* OR psychotic* OR “autism spectrum disorder*” OR ASD OR autism OR autistic* OR depressi* OR “mood disorder*” OR internalizing OR “emotional problem*” OR behavioural OR behavioral OR “behavioral problem*” OR “behavioural problem*” OR affective OR “affective disorder*” OR unipolar OR bipolar OR anxiety OR anxious* OR “eating disorder*” OR mutism OR attachment OR “attachment disorder*” OR phobia* OR anorexia OR bulimia OR ADHD OR “attention deficit hyperactivity disorder*” OR externalizing OR OCD OR “obsessive compulsive disorder*” OR "obsessive-compulsive disorder*" OR “tic-disorder*” OR "tic disorder*" OR "developmental disorder*" OR “substance use disorder*” OR "substance abuse*" OR “substance abuse disorder*” OR “posttraumatic stress disorder*” OR “posttraumatic stress symptom*” OR “post-traumatic stress disorder*” OR “post-traumatic stress symptom*” OR PTSD OR PTSS OR Tourette* OR “neuropsychiatric disorder*” OR “personality disorder*” OR “conduct disorder*” OR “oppositional defiant disorder” OR “ODD”)

MEDLINE (via EBSCOhost)

SU (“Vitamin D*” OR “Vit D*” OR “25-hydroxyvitamin D*” OR “25(OH)D*” OR “25-OH-D*” OR “25 OH D*” OR “1,25-dihydroxyvitamin D*” OR "Vitamin D"[Mesh]) AND (pregnancy OR antenatal OR “ante-natal*” OR “ante natal*” OR antepartum OR “ante-partum*” OR “ante partum*” OR pregnant OR prenatal OR “pre-natal*” OR “pre natal*” OR maternal OR mother* OR gestation* OR utero OR delivery OR perinatal OR “peri natal*” OR “peri-natal*” OR "Pregnancy"[Mesh] OR offspring* OR child* OR infan* OR newborn* OR baby OR babies OR adolesc* OR toddler* OR preschooler* OR “pre-schooler*” OR “pre schooler*” OR “adult*” OR “early life*”OR "Child"[Mesh] OR "Infant"[Mesh] OR "Adult Children"[Mesh]) AND (sera OR biomarker* OR serum OR plasma OR blood OR circulating OR cord OR "Biomarkers"[Mesh]) AND (“psychiatric illness*” OR “psychiatric diagnos*” OR “psychiatric disorder*”OR “mental disorder*” OR “mental health*” OR “mental illness*” OR schizo* OR psychos* OR psychotic* OR “autism spectrum disorder*” OR ASD OR autism OR autistic* OR depressi* OR “mood disorder*” OR internalizing OR “emotional problem*” OR behavioural OR behavioral OR “behavioral problem*” OR “behavioural problem*” OR affective OR “affective disorder*” OR unipolar OR bipolar OR anxiety OR anxious* OR “eating disorder*” OR mutism OR attachment OR “attachment disorder*” OR phobia* OR anorexia OR bulimia OR ADHD OR “attention deficit hyperactivity disorder*” OR externalizing OR OCD OR “obsessive compulsive disorder*” OR "obsessive-compulsive disorder*" OR “tic-disorder*” OR "tic disorder*" OR "developmental disorder*" OR “substance use disorder*” OR "substance abuse*" OR “substance abuse disorder*” OR “posttraumatic stress disorder*” OR “posttraumatic stress symptom*” OR “post-traumatic stress disorder*” OR “post-traumatic stress symptom*” OR PTSD OR PTSS OR Tourette* OR “neuropsychiatric disorder*” OR “personality disorder*” OR “conduct disorder*” OR “oppositional defiant disorder” OR “ODD” OR "Mental Disorders"[Mesh])

EMBASE

#1 AND #2 AND #3 AND #4

#4 'psychiatric illness*':ti,ab OR 'psychiatric diagnos*':ti,ab OR 'psychiatric disorder*':ti,ab OR 'mental disorder*':ti,ab OR 'mental health*':ti,ab OR 'mental illness*':ti,ab OR schizo*:ti,ab OR psychos*:ti,ab OR psychotic*:ti,ab OR 'autism spectrum disorder*':ti,ab OR asd:ti,ab OR autism:ti,ab OR autistic*:ti,ab OR

depressi*:ti,ab OR 'mood disorder*':ti,ab OR internalizing:ti,ab OR 'emotional problem*':ti,ab OR 'behavioral problem*':ti,ab OR behavioural:ti,ab OR behavioral:ti,ab OR 'behavioural problem*':ti,ab OR affective:ti,ab OR 'affective disorder*':ti,ab OR unipolar:ti,ab OR bipolar:ti,ab OR anxiety:ti,ab OR anxious*:ti,ab OR 'eating disorder*':ti,ab OR mutism:ti,ab OR attachment:ti,ab OR 'attachment disorder*':ti,ab OR phobia*:ti,ab OR anorexia:ti,ab OR bulimia:ti,ab OR adhd:ti,ab OR 'attention deficit hyperactivity disorder*':ti,ab OR externalizing:ti,ab OR ocd:ti,ab OR 'obsessive compulsive disorder*':ti,ab OR 'obsessive-compulsive disorder*':ti,ab OR 'tic-disorder*':ti,ab OR 'tic disorder*':ti,ab OR 'developmental disorder*':ti,ab OR 'substance use disorder*':ti,ab OR 'substance abuse*':ti,ab OR 'substance abuse disorder*':ti,ab OR 'posttraumatic stress disorder*':ti,ab OR 'posttraumatic stress symptom*':ti,ab OR 'post-traumatic stress disorder*':ti,ab OR 'post-traumatic stress symptom*':ti,ab OR ptsd:ti,ab OR ptss:ti,ab OR tourette*:ti,ab OR 'neuropsychiatric disorder*':ti,ab OR 'personality disorder*':ti,ab OR 'conduct disorder*':ti,ab OR 'oppositional defiant disorder':ti,ab OR odd:ti,ab

#3 sera:ti,ab OR biomarker*:ti,ab OR serum:ti,ab OR plasma:ti,ab OR blood:ti,ab OR circulating:ti,ab OR cord:ti,ab

#2 pregnancy:ti,ab OR antenatal:ti,ab OR 'ante-natal*':ti,ab OR 'ante natal*':ti,ab OR antepartum:ti,ab OR 'ante-partum*':ti,ab OR 'ante partum*':ti,ab OR pregnant:ti,ab OR prenatal:ti,ab OR 'pre-natal*':ti,ab OR 'pre natal*':ti,ab OR maternal:ti,ab OR mother*:ti,ab OR gestation*:ti,ab OR utero:ti,ab OR delivery:ti,ab OR perinatal:ti,ab OR 'peri natal*':ti,ab OR 'peri-natal*':ti,ab OR offspring*:ti,ab OR child*:ti,ab OR infan*:ti,ab OR newborn*:ti,ab OR baby:ti,ab OR babies:ti,ab OR adolesc*:ti,ab OR toddler*:ti,ab OR preschooler*:ti,ab OR 'pre-schooler*':ti,ab OR 'pre schooler*':ti,ab OR 'adult*':ti,ab OR 'early life*':ti,ab

#1 'vitamin d*':ti,ab OR 'vit d*':ti,ab OR '25-hydroxyvitamin d*':ti,ab OR '25(oh)d*':ti,ab OR '25-oh-d*':ti,ab OR '25 oh d*':ti,ab OR '1,25-dihydroxyvitamin d*':ti,ab

Web of science

TS=("Vitamin D*" OR "Vit D*" OR "25-hydroxyvitamin D*" OR "25(OH)D*" OR "25-OH-D*" OR "25 OH D*" OR "1,25-dihydroxyvitamin D*") AND TS= (pregnancy OR antenatal OR "ante-natal*" OR "ante natal*" OR antepartum OR "ante-partum*" OR "ante partum*" OR pregnant OR prenatal OR "pre-natal*" OR "pre natal*" OR maternal OR mother* OR gestation* OR utero OR delivery OR perinatal OR "peri natal*" OR "peri-natal*" OR offspring* OR child* OR infan* OR newborn* OR baby OR babies OR adolesc* OR toddler* OR preschooler* OR "pre-schooler*" OR "pre schooler*" OR "adult*" OR "early life*") AND TS= (sera OR biomarker* OR serum OR plasma OR blood OR circulating OR cord) AND TS= ("psychiatric illness*" OR "psychiatric diagnos*" OR "psychiatric disorder*"OR "mental disorder*" OR "mental health*" OR "mental illness*" OR schizo* OR psychos* OR psychotic* OR "autism spectrum disorder*" OR ASD OR autism OR autistic* OR depressi* OR "mood disorder*" OR internalizing OR "emotional problem*" OR behavioural OR behavioral OR "behavioral problem*" OR "behavioural problem*" OR affective OR "affective disorder*" OR unipolar OR bipolar OR anxiety OR anxious* OR "eating disorder*" OR mutism OR attachment OR "attachment disorder*" OR phobia* OR anorexia OR bulimia OR ADHD OR "attention deficit hyperactivity disorder*" OR externalizing OR OCD OR "obsessive compulsive disorder*" OR "obsessive-compulsive disorder*" OR "tic-disorder*" OR "tic disorder*" OR "developmental disorder*" OR "substance use disorder*" OR "substance abuse*" OR "substance abuse disorder*" OR "posttraumatic stress disorder*" OR "posttraumatic stress symptom*" OR "post-traumatic stress disorder*" OR "post-traumatic stress symptom*" OR PTSD OR PTSS OR Tourette* OR "neuropsychiatric disorder*" OR "personality disorder*" OR "conduct disorder*" OR "oppositional defiant disorder" OR "ODD")

Table S1. Quality assessment tables using JBI for cohort and case control studies

For cohort studies

SN	Authors	Were the two groups similar and recruited from the same population?	Were the exposures measured similarly to assign people to both exposed and unexposed groups?	Was the exposure measured in a valid and reliable way?	Were confounding factors identified?	Were strategies to deal with confounding factors stated?	Were the groups/participants free of the outcome at the start of the study (or at the moment of exposure)?	Were the outcomes measured in a valid and reliable way?	Was the follow up time reported and sufficient to be long enough for outcomes to occur?	Was follow up complete, and if not, were the reasons to loss to follow up described and explored?	Were strategies to address incomplete follow up utilized?	Was appropriate statistical analysis used?	Overall appraisal: Include, Exclude, Seek further info	Comments (Including reason for exclusion)
1	Allen et al., 2013 [16]	Yes	Yes	Yes	Yes	Yes	Yes	Yes	Yes	Unclear	Yes	Yes	Include	
2	Chawla et al., 2019 [39]	Yes	Yes	Yes	Yes	Yes	Yes	Yes	No	No	No	Yes	Include	
3	Chu et al., 2022 [44]	Yes	Yes	Yes	Yes	Yes	Yes	Yes	Yes	Yes	No	Yes	Include	
4	Daraki et al., 2018 [22]	Yes	Yes	Yes	yes	Yes	Yes	Yes	No	Yes	Yes	Yes	Include	
5	Fernell et al., 2015 [25]	No	Yes	Yes	No	No	Yes	Yes	Yes	Unclear	No	No	Include	
6	López-Vicente et al., 2019 [37]	Yes	Yes	Yes	Yes	Yes	Yes	Yes	Yes	No	No	Yes	Include	

7	Ma et al., 2021 [19]	Yes	Yes	Yes	Yes	Yes	Yes	Yes	No	No	No	Yes	Include	
8	Morales et al., 2015 [28]	Yes	Yes	Yes	Yes	Yes	Yes	No	No	Unclear	No	Yes	Include	
9	Mossin et al., 2017 [29]	Yes	Yes	Yes	Yes	Yes	Yes	Yes	No	Unclear	No	Yes	Include	
10	Strøm et al. 2014 [31]	Yes	Yes	Yes	Yes	Yes	Yes	Yes	Yes	Yes	Yes	Yes	Include	
11	Sullivan et al., 2013 [33]	Yes	Yes	Unclear	Yes	Yes	Yes	Unclear	Unclear	Unclear	Unclear	Yes	Include	
12	Vinkhuyzen et al., 2017 [34]	Yes	Yes	Yes	Yes	Yes	Yes	Yes	Unclear	Yes	Yes	Yes	Include	
13	Vinkhuyzen et al., 2018 [35]	Yes	Yes	Yes	Yes	Yes	Yes	Yes	Yes	Yes	Yes	Yes	Include	
14	Wang et al., 2020 [36]	Yes	Yes	Yes	Yes	Yes	Yes	Unclear	Yes	Yes	Unclear	Yes	Include	
15	Whitehouse et al., 2013 [18]	Yes	Yes	Yes	Yes	Yes	Yes	Unclear	Yes	No	Yes	Yes	Include	
16	Whitehouse et al., 2012 [17]	Yes	Yes	Yes	Yes	Yes	Yes	Yes	Yes	No	Unclear	Yes	Include	

For case-control studies

S. n	Authors	Were the groups comparable other than the	Were cases and controls matched appropriately?	Were the same criteria used for identificatio	Was exposure measured in a standard	Was exposure measured in the same	Were confounding factors identified?	Were strategies to deal with confoundin	Were outcomes assessed in a	Was the exposure period of interest long	Was appropriate statistical analysis used?	Overall appraisal: Include, Exclude, Seek	Comments (Including reason for exclusion)

		presence of disease in cases or the absence of disease in controls?		n of cases and controls?	, valid and reliable way?	way for cases and controls?		g factors stated?	standard , valid and reliable way for cases and controls?	enough to be meaningful ?		further info	
1	Chen et al., 2016 [20]	Yes	Yes	Yes	Yes	Yes	Yes	Yes	Yes	Yes	Yes	Include	
2	Eyles et al., 2018 [24]	Yes	Yes	Yes	Yes	Yes	Yes	Yes	Yes	Yes	Yes	Include	
3	Gustafsson et al., 2015 [26]	Yes	Yes	Yes	Yes	Yes	Yes	Yes	Yes	Yes	Yes	Include	
4	McGrath et al., 2003 [40]	Yes	Yes	Yes	Yes	Yes	No	No	Yes	Yes	Yes	Include	
5	McGrath et al., 2010 [27]	Yes	Yes	Yes	Yes	Yes	Yes	Yes	Unclear	Yes	Yes	Include	
6	Schmidt et al., 2019 [41]	Yes	Unclear	Yes	Yes	Yes	Yes	Yes	Unclear	Yes	Yes	Include	
7	Sourander et al., 2021 [30]	Yes	Yes	Yes	Yes	Yes	Yes	Yes	Yes	Yes	Yes	Include	
8	Sucksdorff et al., 2021 [32]	Yes	Yes	Yes	Yes	Yes	Yes	Yes	Yes	Yes	Yes	Include	
9	Windham et al.2019 [42]	Yes	Unclear	Yes	Yes	Yes	Yes	Yes	Yes	Yes	Yes	Include	
10	Windham et al., 2020 [43]	Yes	Unclear	Yes	Yes	Yes	Yes	Yes	Yes	Yes	Yes	Include	

11	Wu et al., 2018 [21]	Yes	Yes	Yes	Yes	Yes	Yes	Yes	Yes	Yes	Yes	Include	
12	Lee et al., 2021 [38]	Yes	Yes	Yes	Yes	Yes	Yes	Yes	Yes	Yes	Yes	Include	
13	Egorova et al., 2020 [23]	Yes	Yes	Yes	Yes	Yes	Yes	Yes	Yes	Unclear	Yes	Include	

Table S2. Findings of the reviewed studies by each outcome. The studies are colour marked by the measurement time of vitamin D.

Outcome	Positive findings	Mixed findings	Null findings
ADHD	Chu et al., 2017 [44]		Chu et al., 2017 [44]
	Sucksdorff et al., 2021 [32]		Gustafsson et al., 2015 [26]
			Strøm et al., 2014 [31]
ADHD symptoms	Daraki et al., 2018 [22]		López-Vicente et al., 2019 [37]
	Morales et al., 2015 [28]		Ma et al., 2021 [19]
	Mossin et al., 2017 [29]		
ASD	Chen et al., 2016 [20]	Lee et al., 2021 [38]	Egorova et al., 2020 [23]
	Lee et al., 2021 [38]	Schmidt et al., 2019 [41]	Vinkhuyzen et al., 2017 [34]
	Fernell et al., 2015 [25]	Windham et al., 2019 [42]	
	Sourander et al., 2021 [30]	Windham et al., 2020 [43]	
	Vinkhuyzen et al., 2017 [34]		
	Wu et al., 2018 [21]		
ASD symptoms	Vinkhuyzen et al., 2018 [35]	Chawla et al., 2019 [39]	López-Vicente et al., 2019 [37]
	Vinkhuyzen et al., 2018 [35]		Whitehouse et al., 2013 [18]
Depressive disorder			Strøm et al., 2014 [31]
Depressive symptoms			Wang et al., 2020 [36]
Eating disorder symptoms		Allen et al., 2013 [16]	
Schizophrenia or schizoaffective disorder	Eyles et al., 2018 [24]		McGrath et al., 2003 [40]
	McGrath et al., 2010 [27]		
Psychotic experiences			Sullivan et al., 2003 [33]
Behavioural or emotional difficulties	Daraki et al., 2018 [22]	Chawla et al., 2019 [39]	López-Vicente et al., 2019 [37]
			Whitehouse et al., 2012 [17]

Maternal sera at first or second trimester
Maternal sera at third trimester, from cord or neonatal samples
Maternal sera at any time of pregnancy

Figure S1. Studies and the age of offspring by the time when outcomes were assessed. Blue box indicates exact assessment time point and grey box indicates age range.

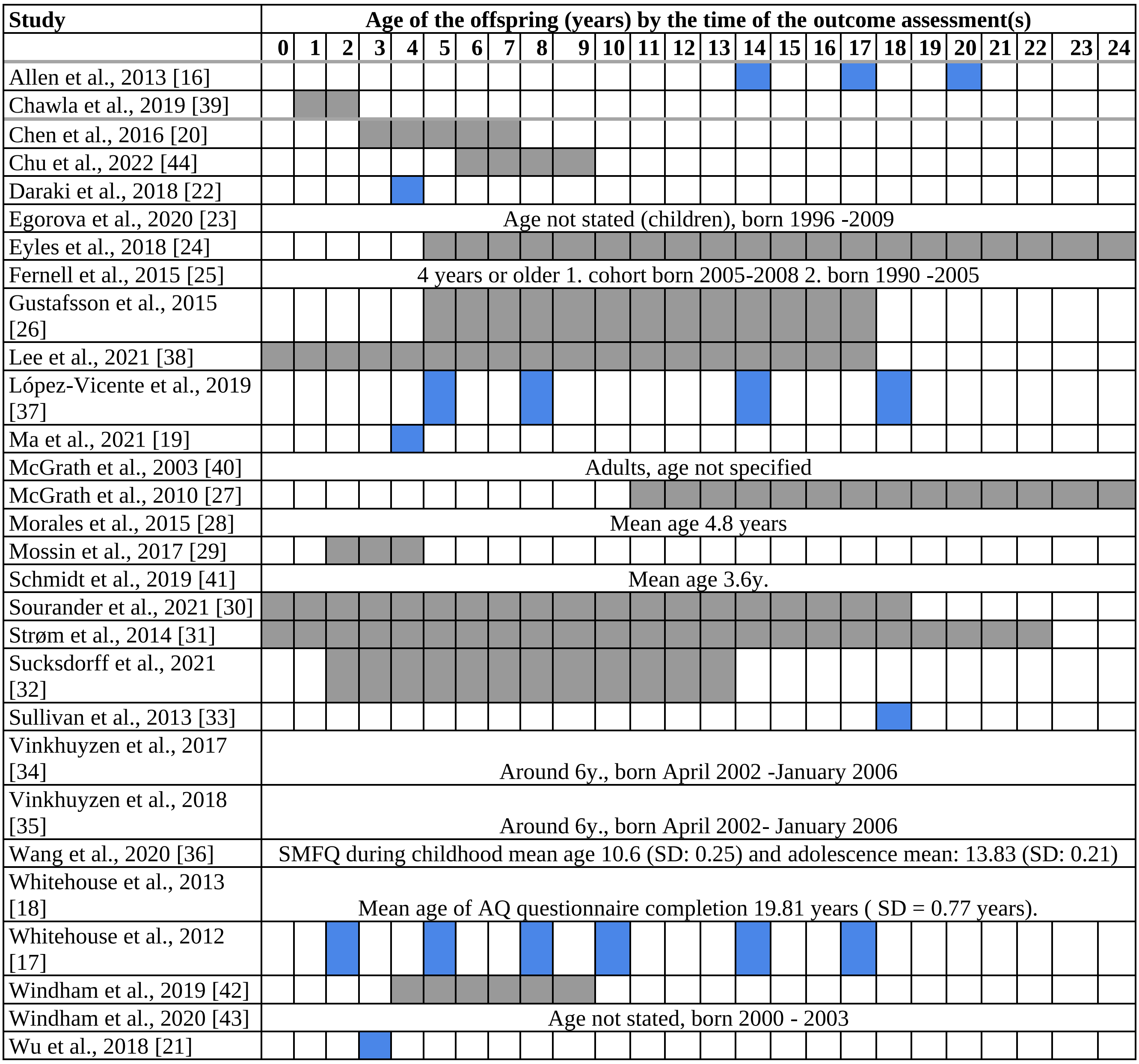

Supplement: Supplementary file 1 [file ijms-24-00063-s001.zip › ijms-2059037-supplementary.pdf]
